# An Inquiry into the Nature of Sleep and Death, &c.

**Published:** 1834-10-01

**Authors:** 


### An Inquiry into the Nature of Sleep and Death, with a view to ascertain the more immediate Causes of Death, and the better Regulation of the Means of obviating them; being the concluding part of the Author's Experimental Inquiry into the Laws of the Vital Functions. (Re-published from the Philosophical Transactions.) By *A. P. W. Philip*, M.D. &c. &c. 8vo. pp. 254.

Our first glance over the title-page of this volume engendered a hope that Dr. Philip had discovered some means of baffling the grim tyrant Death, by depriving him of the weapons by which he cuts down man at all periods of existence. A more close examination, both of the title and the body of the work, has lessened our hopes, and left us in possession of the facts, that diseases and even disorders of the vital organs and functions, carry us off *before* the age of three score years and ten—and that, even if we are fortunate enough to escape sickness, TIME itself will moulder us into dust *after* that epoch! If ever man deserved immortality, Dr. Philip is that man; for he has studied the "laws of the vital functions," till, we should imagine, he has them all by heart. But if our worthy and indefatigable author has not been able by any experiments on animals, to secure himself from the grave, he has done enough to transmit his name and his fame to posterity—(which is all that the most talented can do, or the most ambitious expect)—and that too notwithstanding the Ulyssean stratagems of Prout, or the Ajax-like lounges of Earle against the "laws" laid down by Dr. Philip.

As most of the papers in this volume have been long before the public, it is not our intention to notice more than the last two chapters, on sleep and death. Nor shall we go very minutely into these. The former subject is a drowsy, and the latter is a melancholy one. Most people, indeed, would rather enjoy the refreshment of a sound sleep, than listen to the most philosophical discussion on its nature and causes. However, as physicians and philosophers must have many sleepless hours, they may as well amuse themselves with a discussion on this, as on any other subject. Dr. Philip's long dissertation on sleep may be brought into a very narrow compass. There is no organ or structure in the body, neither heart, arteries, muscles, or glands, that can carry on uninterrupted action—Dr. Philip to the contrary notwithstanding—and, therefore, the sensorial functions require their period of rest, as well as those of the stomach or any other part. The brain is tired, during the day, by the reception of sensations and the exertion of reflections, volitions, &c. and at night we sleep. Dr. Philip maintains that the arteries are in *perpetual* action. We deny it, nor would a thousand experiments on frogs or rabbits convince us of this perpetuity. Dr. Philip admits that the heart appears to have a periodical relaxation, and cessation of its proper stimulus, the blood. Now the heart is never free from the presence of blood, as Dr. Philip must know. The ventricle never empties itself entirely. But as the systole occupies only about a third part of the time occupied by the diastole, it follows that the muscular fibres of the heart are sixteen out of the twenty-four hours in a state of inaction. This is about the period of rest which is enjoyed by the voluntary muscles. The same argument applies to the arteries. If we recollect right, Dr. Philip maintained, in opposition to Dr. Parry, that the arteries contracted and dilated like the heart, though in different rhythms. If this be the case, they have their alternations of labour and repose, like other muscular structures. The muscles of respiration, we need not say, have the same proportions of rest. Even the secreting organs, as the liver and kidneys, which appear to be more perpetually in action than almost any other parts of the human frame have, in reality, alternate labour and repose. Secretion may never, perhaps, entirely cease; but it is periodically in activity and inactivity, comparatively, which answers the same purpose. As to the veins, it will not be said that they are in perpetual action. During the greater part of their time, they are merely passive hydraulic machines, or tubes to convey the blood from one part to another.

Dr. Philip maintains that that part of the brain which supplies nervous energy to the vital organs, as the heart, arteries, &c. is in a state of perpetual or uniform excitement. This may be so; but, if we are correct in shewing that the functions of these organs are in alternate action and repose, we do not see why the brain itself should not be similarly situated? We shall give Dr. Phillip's conclusions, however, in his own words, as containing the pith of the paper.

"From a review of the whole of the facts which have been laid before the Society, it appears,—

1. That in the brain and spinal marrow alone reside the active parts of the nervous system.

2. That the law of excitement in the parts of these organs, which are associated with the nerves of sensation and voluntary motion, is uniform excitement followed by proportional exhaustion, which, when it takes place to such a degree as to suspend their usual functions, constitutes sleep; all degrees of exhaustion which do not extend beyond them and the parts associated with them, being consistent with health.

3. That the law of excitement in those parts of the brain and spinal marrow which are associated with the vital nerves is also uniform excitement, but which is only, when excessive, followed by any degree of exhaustion, no degree of which is consistent with health.

4. That the vital, in no degree partaking of the exhaustion of the sensitive system in sleep, only appears to do so from the influence of the latter on the function of respiration, the only vital function in which these systems co-operate, in consequence of which its organs, without being in any degree debilitated, are less readily excited.

5. That the law of excitement of the muscular fibre, with which both the vital and sensitive parts of the brain and spinal marrow are associated, is interrupted excitement, which, like the excitement of the vital parts of these organs, is only, when excessive, followed by any degree of exhaustion. And

6. That the nature of the muscular fibre is every where the same, the apparent differences in the nature of the muscles of voluntary and involuntary motion depending on the differences of their functions, of their relation to the brain and spinal marrow, and of the circumstances in which they are placed." 150.

Dr. Philip has appended some observations on dreaming, which have nothing particularly novel in them. Although the sensorial powers of the brain become so exhausted that sleep ensues, it does not follow that they are so completely exhausted as not to receive impressions, though indistinctly. It is these faint impressions, from within or without, that cause dreaming. These causes are chiefly from irritation in the digestive organs, as every one who has eaten a heavy supper—or who has weak powers of digestion, must well know. The silence and quietude that generally prevail during sleep tends, also, to promote the perception of sensations then, which otherwise would pass unheeded even when we are wide awake, and when the mind is employed in the avocations of the day. The rapidity of the operations of the memory and imagination, during sleep and in the act of dreaming, is most curious and unaccountable, and so are the incongruities which then occur, and which appear perfectly natural and correct to the dreamer. Dr. Philip endeavours to explain these phenomena in the following manner, but with what success we shall not decide.

" It seems greatly to influence the phenomena of dreaming, that in order to favour the occurrence of sleep, and thus as far as we can prevent unnecessary exhaustion, means are always employed at its accustomed times, to prevent, as much as possible, the excitement of the external organs of senses, and consequently those parts of the brain associated with them. This renders us the more sensible to causes of excitement existing within our own bodies, while, by the inactivity of the parts of the brain which are associated with those organs, we are deprived of the usual control over such parts of the mental functions as are thus excited ; the effect of which is greatly increased by the rapidity of the operations of the memory and imagination, when not restrained by some of the various means employed for that purpose in our waking hours. These are often objects of the senses, as written language, diagrams, sounds, and sometimes even objects of touch ; but the most common is the mere use of words, independently of any object presented to our senses.

Any one may easily perceive how difficult it is to pursue a train of reasoning without this means of detaining his ideas for the purpose of steadily considering them and comparing them together. Now, in sleep, in consequence of the excitement of the brain being so partial, we are deprived of all these means; and our ideas pass with such rapidity as precludes all consideration and comparison. Our conceptions, therefore, are uncorrected by experience, and we are not at all surprised at the greatest incongruities. Why should we be surprised at our moving through the air, when we are not aware that we have not always done so? The mind of the dreamer differs from that of the infant in having been variously impressed, and therefore in the capability of having its impressions recalled. But it is only as far as it is excited, that any impression can be recalled. With this exception it is as void of the results of experience as the mind of the infant; and therefore, in its partial excitement, of the means of correcting any particular train of ideas suggested. In general, there is neither time nor means for reflection, and, consequently, there can neither be doubt nor hesitation.

Such is the rapidity of our thoughts in dreaming, that it is not uncommon for a dream, excited by the noise that awakes us, and which, therefore, must take place in the act of awaking, to occupy, when put into words, more than fifty times the space in the relation. It is a good illustration of what is here said, that when we dream that we are conversing, and thus obliged to employ words, the usual incongruities of dreaming do not occur. The ideas are sufficiently detained to enable us to correct the suggestions of the imagination. No man ever dreamt that he was telling another that he had been flying through the air.

Thus the peculiarities of dreaming arise from the partial operation of the causes of disturbance, and some of the sensitive parts of the brain being capable of excitement without disturbing the others; and thus it is that the more near we are to awaking, the more rational our dreams become, all parts of the brain beginning to partake of the excitement; which has given rise to the adage, that morning dreams are true." 153.

### Nature of Death.

Dr. Philip does not pretend to rend the veil that covers the nature of death in a metaphysical sense of the word. There are some expressions in the opening of this paper which we can hardly agree in. Dr. Philip says that all our knowledge is not acquired, through the medium of our senses, after we are born. "We come into the world with *knowledge* essential to our existence." How does our author support this position? Because, says he, "the infant *knows* as well how to *breathe* and suck as the adult, and these acts depend as much on *mental* operations as those which are the result of experience." Indeed! the act of breathing depends on *mental* operations, when that same act goes on in the profoundest sleep, or in deadly apoplexy, when no mental operation goes on at all! Really, we are astonished that the author of a treatise on the vital functions should assert that the breathing of an infant, when first launched into this world, depends on mental operations. If he had said on *cerebral* operations, there might have been some truth in the assertion; but we maintain that breathing has nothing to do with mental operations—excepting when we are blowing a fire or a flute, which will hardly be called ordinary respiration. We do not deny that the child is born with a few *instincts*, chiefly the art of *sucking*, and that is almost the only one. This instinct or impulse has no analogy to acquired knowledge at all. The instinct cannot be resisted either by infant or animal. It is as much an *involuntary* action as breathing itself. The child will suck when asleep. We believe with Locke, and many others, who have not been deficient in human understanding, that the mind, or rather its organ, is a complete blank when we are born, and that all knowledge is written there afterwards by sensation and reflection. This last may combine and multiply knowledge, but all the raw materials must come through the senses. We do not consider the instinctive impulse to sucking as any species of knowledge. The smell of the mother's milk excites the olfactory and gustatory nerves of the infant, in the same way that dust would excite coughing, or snuff sneezing. There would be no great pretence to knowledge in either of these processes, we calculate. Dr. Philip gives us another piece of information, which we confess we were quite ignorant of before. Speaking of this same new-born infant, he says—" he perceives his wants, and he knows how to relieve them." To this we demur. We believe that the infant feels some few of his wants, as the want of food; but we also believe, and indeed we are certain, that he does *not* know how to relieve himself, and, therefore, he squalls out most lustily, till his mother or nurse supplies him. When an infant feels cold, does he know how to relieve himself by additional clothing? Really we never heard such loose, not to say erroneous, reasoning, from any one professing to be an experimental philosopher, as we find in this book.

We find it utterly impossible to give any thing like a connected view of the paper under consideration, since it refers at every page, and almost in every paragragh, to " my Inquiry into the Laws of the Vital Functions"—or " the Papers which I had the honour of publishing in the Philosophical Transactions"—all or any of which we have neither time nor inclination to peruse or re-peruse. We are not certain, indeed, that we can always comprehend our author; and in such cases we must adopt the language and conduct of Parliamentary reporters, who, when they cannot hear a word of the orator, inform their readers that " they understood him to say," &c. &c. We understand Dr. Philip, then, to hold that, in the more perfect animals, there are three distinct classes of functions—the sensorial, the nervous, and the muscular, not directly dependent on each other, but only through the influence of their respective organs, the destruction of any one class soon leading to the destruction of the others. The sensorial functions, constituting the sensitive system, and connecting man with the external world, first fails, in natural death, the nervous and muscular systems continuing for a time, but ultimately failing, " in consequence of the failure of respiration, the only vital function to which the co-operation of the sensorial power is necessary." Thus, after all our learning and science, we come to the old conclusion, that " man dies for want of breath." Another conclusion to which the light of science has led us, has long been come to without science at all—namely, that death is only an everlasting sleep.

" The state which immediately precedes the last act of dying then, according to the common acceptation of the term, and sleep, depend on a failure of function in the same organs. In what, then, consists the difference of these states? The most evident is, that the one is a temporary, the other a final failure; and it will appear, that in the only death which can strictly be called natural, the state of the sensitive system which immediately precedes death differs from its state in sleep in no respect but in degree." 163.

Our author asserts, and the assertion is highly consolatory, that " we are not necessarily born to suffering." Is this quite consistent with Scripture? " All natural states (says Dr. Philip) with the exception of child-bearing (and, in its most natural state, even this is hardly an exception), are more or less pleasurable." Does the infant cut its teeth, even in the most natural state, without some suffering? We apprehend that it does not. Those who dread death on account of the *agonies* which are usually considered as its attendant, may derive consolation from the following passage.

"The death of old age, therefore, is literally the last sleep, uncharacterized by any peculiarity. The general languor of the functions in the last waking interval is attended with no peculiar suffering, and the last sleep commences with the usual grateful feelings of repose, the last feelings experienced; for with what takes place after them, the feelings, being suspended, have no concern." 166.

This pleasing picture (and we believe it to be a correct one) of the last scene in man's "strange eventful history," is darkened by the reflection that, in civilized life, we rarely see an instance of natural death, as above-described—at least at the natural period of human existence. Dr. Philip informs us that, "of the various instances of death which he has witnessed, there was not one that could be regarded as wholly the effect of age." We have seen more than one instance, where death appeared to be the effect of very advanced age, and where there was no cognizable or tangible disease. We think that when a man dies, worn out by 80 or 90 years, he may fairly be said to die the death of nature. The late Sir Gilbert Blane died in his 85th year, and we are not aware that he laboured under any disease. It is, however, very rare to find all the organs so balanced in strength, that no one gives way under the numerous morbific causes to which we are exposed before the threescore years and ten. Still, even when we are brought to the grave by premature disease, the last scene is the death of nature after all. Look at the individual dying of fever, consumption, apoplexy, &c.; we see him lie for hours in a state of stupor, the sleep of death, unconscious of surrounding friends, and insensible to sufferings if they actually exist. It is long before the act of dying, that the individual suffers from the effects of disease. When the malady has brought him to the last act of the drama, the sufferings are over, and he sleeps, or perchance dreams, away the last hours of his existence. But we must draw our notice of Dr. Philip's work to a conclusion with the following extract, the observations in which are very rational and correct.

"The approach of death, if we are aware of it, must always be more or less impressive, not only because we are about to undergo an unknown change, but are leaving all that has hitherto interested and been grateful to us. Even here, however, for the most part, the laws of our nature are merciful. Most diseases of continuance (for we shall find there are some exceptions) not only gradually impair our sensibility, but alter our tastes. They not only render us less sensible to all impressions, but less capable of enjoying as far as we are still sensible to them. The sight of a feast to a man who has lost his appetite is disgustful, and a similar change takes place in a greater or less degree with respect to all other means of enjoyment.

These circumstances constitute a great part of the difference of our feelings with respect to what, in common language, is called a violent and a natural death. In the latter, as far as the sensibility is impaired, we are more or less in the state of old age, and, in addition to this change, our tastes are perverted. By these means, the relish for life is in a great degree destroyed before we lose it. Thus in disease, the most timid often meet death with composure, and sometimes, as I have repeatedly witnessed, with pleasure. I have even known the information that the danger was passed, received only with expressions of regret." 187.

We heartily wish that Dr. Philip would treat us to a good sound dish of original information, instead of hashing up, in so many new shapes, the fragments of former meals, some of which have got stale, and others not over-wholesome. Let Dr. Philip ponder on what the reviewer of his works, in the last Number of the Edinburgh Medical and Surgical Journal, says on this subject:—

"In almost every page, the author's *peculiar* opinions, physiological and pathological, are *inextricably* mixed with the more immediate object of the treatise, so that, to enter into any discussion on the subject would be, in fact, to review the author's former works."*

We are not, therefore, singular in our strictures on this hashing system of bibliography. If Dr. P. was an ordinary writer, and incapable of producing original works, there might be some excuse for compilation; but he has not this excuse to make, and would not, we apprehend, be very fond of making it, if true.

* Ed. Journal, July, 1834, p. 178.

